# Advances of Carbon-Based Materials for Lithium Metal Anodes

**DOI:** 10.3389/fchem.2020.595972

**Published:** 2020-10-21

**Authors:** Kaikai Tang, Jun Xiao, Xiao Li, Dandan Wang, Mengqi Long, Jun Chen, Hong Gao, Weihua Chen, Chuntai Liu, Hao Liu

**Affiliations:** ^1^Joint International Laboratory on Environmental and Energy Frontier Materials, School of Environmental and Chemical Engineering, Shanghai University, Shanghai, China; ^2^State Key Laboratory of Advanced Special Steel, Shanghai Key Laboratory of Advanced Ferrometallurgy, Shanghai University, Shanghai, China; ^3^Key Laboratory of Materials Processing and Mold (Zhengzhou University), Ministry of Education, Zhengzhou, China; ^4^Centre for Clean Energy Technology, Faculty of Science, School of Mathematical and Physical Sciences, University of Technology Sydney, Sydney, NSW, Australia

**Keywords:** composite anodes, current collectors, additives, carbon-based materials, lithium metal anodes, battereis

## Abstract

Lithium metal with high theoretical specific capacity (3,860 mAh g^−1^), low mass density, and low electrochemical potential (−3. 040 V vs. SHE) is an ideal candidate of the battery anode. However, the challenges including dendrite propagation, volume fluctuation, and unstable solid electrolyte interphase of lithium metal during the lithium plating impede the practical development of Lithium metal batteries (LMBs). Carbon-based materials with diverse structures and functions are ideal candidates to address the challenges in LMBs. Herein, we briefly summarize the main challenges as well as the recent achievements of lithium metal anode in terms of utilizing carbon-based materials as electrolyte additives, current collectors and composite anodes. Meanwhile, we propose the critical challenges that need to be addressed and perspectives for ways forward to boost the advancement of LMBs.

## Introduction

Since Sony commercialized lithium-ion batteries (LIBs) in 1991, rechargeable LIBs have been successfully applied to portable electronics and electric vehicles (Yang G. et al., 2019; Yang T. et al., 2019; Lee et al., 2020; Maroufi et al., 2020; Pellow et al., 2020). However, the constrained energy density of the traditional LIBs based on intercalation chemistry are unable to meet the ever-growing requirement of high-energy-density batteries. Lithium metal batteries (LMBs) with high capacity and high energy density have attracted numerous attentions. Compared with commercial LIBs, LMBs employ metallic Li as an anode, which is based on a continuous plating/stripping mechanism, contributing to higher energy output (Chen C. et al., 2019; Zhang T. et al., 2019; Widijatmoko et al., 2020; Ye et al., 2020). Lithium metal anodes are known as the “Holy Grail” electrodes due to the unique advantages, such as the lowest density among metals, high theoretical specific capacity (3,860 mAh g^−1^) and the lowest electrochemical potential (−3.040 V vs. standard hydrogen electrode) (Xie et al., 2019; Shi et al., 2020; Zhang Q. et al., 2020; Zhou Y. et al., 2020). Moreover, when LMBs collocate with high capacity cathodes, such as sulfur (S) and oxygen (O_2_), it can achieve excellent specific energy and be regarded as promising next-generation energy storage systems beyond LIBs and other storage systems (Hong et al., 2019; Sloop et al., 2019; Xiao et al., 2019; Gan et al., 2020; Guo et al., 2020; Li W. T. et al., 2020). Unfortunately, the development of LMBs is hindered by the inevitable shortcomings of lithium metal anode, including dendrite propagation, volume fluctuation, and unstable solid electrolyte interphase (SEI), originating from the high chemical reactivity and “hostless” nature of lithium metal during the plating/stripping process (Li Q. et al., 2018; Li Z. et al., 2020; Pan et al., 2020).

Carbon-based materials with good electrical and thermal conductivity, excellent structural stability, as well as high surface area and abundant surface functional groups have been extensively applied in numerous research fields (Liu et al., 2017; Yan et al., 2019; Tian et al., 2020). Even more noteworthy is that carbon-based materials offer a versatile platform to construct new architectures with interconnected porosity and active sites (Ge et al., 2018; Zhang Z. et al., 2018a; Zhang W. et al., 2019). The surface chemistry of carbon-based materials can be easily modified to improve the binding properties. Consequently, doping or co-doping with different heteroatoms (N, S, P) (Wei et al., 2019; Liu et al., 2020; Ma et al., 2020; Zhang D. M. et al., 2020) makes the carbon-based materials exhibit further enhancement of the electrocatalytic activity. Owing to these diversified functions of carbon-based materials, they have been intensively used to ameliorate the challenges of LMBs (Zuo et al., 2018; Xue et al., 2019; Huang et al., 2020).

Although there are many reported reviews on LMBs, very few reports that specifically describe carbon-based materials in addressing the challenges in LMBs. This review provides a comprehensive overview of the application of carbon-based materials in improving the performance of lithium metal anodes. In this review, we focus on the main challenges faced by lithium metal anodes and emphasize the effective strategies employing carbon-based materials as electrolyte additives, current collectors and composite anodes to achieve high performance lithium metal anodes. We believe that this review is informative and could offer some new insights for this exciting area.

## Challenges of Lithium Metal Anodes

**Figure 1:**
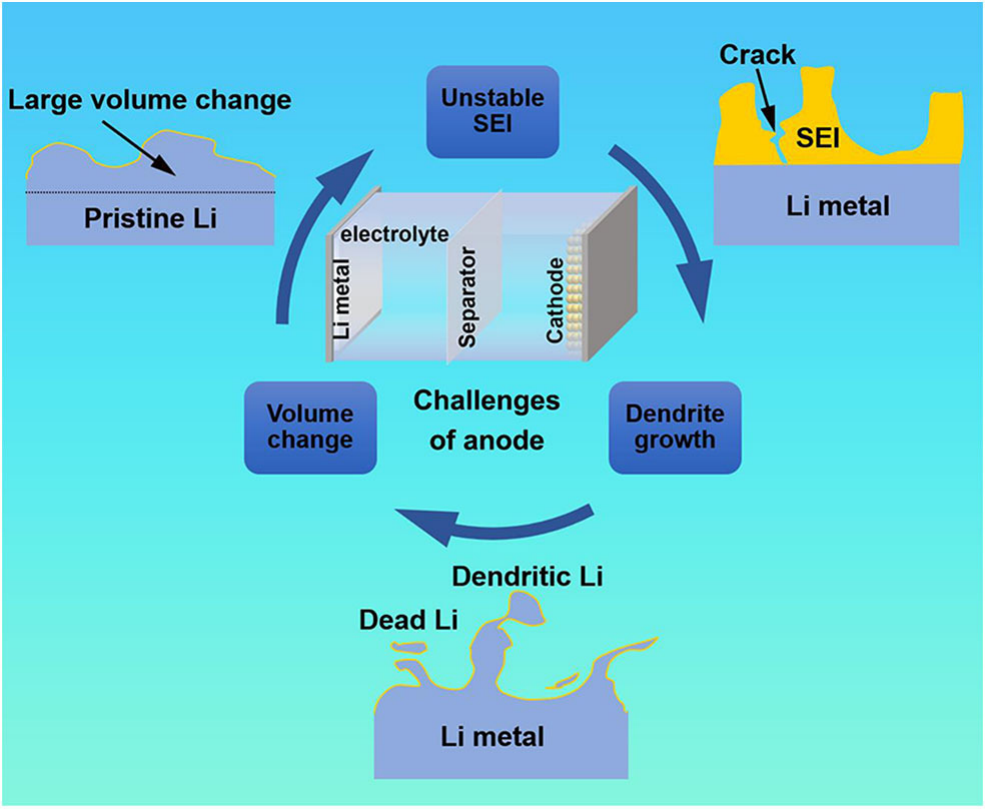
Schematic diagram of the challenge of anode for LMBs.

The LMBs were born in the 1970's. However, they have almost stagnated since then, lagging far behind the development of LIBs. As we all know, lithium metal is very active and can react with almost all organic electrolytes to form a SEI (Figure 1). An ideal SEI film should be electrically insulating and ionically conductive. Moreover, it should have good chemical and mechanical stability to withstand the volume change and protect the lithium metal from further exposure to the electrolyte. Nevertheless, due to the “hostless” property and unevenness of lithium metal surface, Li^+^ preferentially deposits at the tips, resulting in uneven deposition of lithium and the formation of dendrites during the repeatedly stripping and plating process. The dendrites inevitably cause the electrode volume changes, which lead to the destruction of SEI film and then exposure of fresh lithium metal to the electrolyte. This is followed by continuous side reactions until the electrolyte is depleted, which have a negative impact on the Coulomb efficiency (CE). At the same time, the aforementioned process can in turn exacerbate the dendrite growth.

Eventually, there are two possibilities of the uncontrolled dendrites. Firstly, the dendrites may fall off from the anode surface to form “dead Li,” causing the loss of active materials and greatly reduce the utilization of Li. Secondly, the dendrites may pierce the separator to reach the cathode, causing a short circuit. These intrinsic challenges of lithium metal lead to many undesirable drawbacks such as unstable SEI, severe volume change and uncontrolled dendrite growth during electroplating/stripping in the rechargeable LMBs, which have been hindered their practical applications over the past 40 years (Zhang R. et al., 2017).

## Application of Carbon-Based Materials in Lithium Metal Anodes

So far, tremendous strategies have been proposed to suppress the dendrites growth. These strategies can be classified into the following categories: (1) electrolyte manipulation, including modification of additives in the electrolyte (Zhang X. Q. et al., 2017; Huang et al., 2018; Li X. et al., 2018), and adoption of solid or polymer electrolytes (Aldalur et al., 2018; Girard et al., 2019; Huo et al., 2020; Hu Z. et al., 2020); (2) SEI engineering (Li C. et al., 2019; Yan et al., 2020), such as artificial SEI film (Zhu et al., 2019); (3) electrode structure design (Zhang Y. et al., 2018, 2019).

Carbon-based materials with unique physical and chemical properties can act as electrolyte additives to inhibit the dendrite growth. In addition, the various structures, high surface area and flexibility enable the carbon-based materials to be as hosts to accommodate the volume change (Jorge et al., 2019; Nan et al., 2019; Zhang X. et al., 2020).

### Carbon-Based Materials to Construct Composite Anodes

On account of the intrinsic “hostless” of lithium metal, the lithium deposits on the planar electrode will undergo severe volume changes, resulting in continuous fracture and regeneration of the SEI film. Unstable SEI film will seriously reduce the lifespan of the battery. Therefore, confining the lithium metal in scaffold is an effective way to accommodate the volume change (Yang et al., 2018; Pei et al., 2019; Tang et al., 2020). The as-prepared structured composite anode can regulate the Li^+^ deposition and induce dendrite-free morphology, thereby achieving a high capacity and long cycle life lithium metal anode (Wang et al., 2017; Zhang R. et al., 2018a). The 3D porous scaffold with multiple ion and electron transfer paths and high surface area is often employed as host for lithium metal. Moreover, the current density can be dissipated by this 3D conductive porous scaffolds, resulting in a lower local current density (Chazalviel, 1990; Zhou T. et al., 2020), which can effectively control the growth rates of lithium dendrites.

The 3D porous scaffolds, such as copper foam (Yang et al., 2018; Yue et al., 2019b), nickel foam (Huang X. et al., 2019; Sun et al., 2019), mesoporous carbon (Zhang S. J. et al., 2019; Jeong et al., 2020), and carbon nanotube sponge (Yang G. et al., 2019), can accommodate infinite volume expansion and suppress dendrite growth during repeated electroplating/stripping processes. Besides, the composite electrode, assembled with a carbon-based skeleton, has good flexibility and can be used on wearable electronic devices. Nevertheless, most hosts need to be coated with a lithiophilic layer because of their poor lithiophilicity and the requirements of constructing a composite anode. Unlike metal frameworks that require complex processes to enhance lithiophilicity, carbon-based scaffolds have very significant advantages in practical applications. For example, some lithiophilic functional groups can be introduced into the most carbon hosts by facile surface chemistry approaches. Additionally, carbon-based materials with low density enable the high practical energy density batteries.

Tao et al. reported a simple surface ozonolysis and ammoniation treatment strategy to tune the lipophilicity of carbon scaffold (Tao et al., 2020). The flexible and lithiophilic carbon film (CF) is composed of multiple layered interwoven nanofibers. Due to the good mechanical strength and thermal stability, CF can be easily wetted by molten lithium to form a stable Li@CF composite anode. The as-prepared Li@CF composite electrode could deliver a high practical capacity of 3,222 mAh g^−1^ and behave a good rate performance. In addition, no obvious dendrites have been observed in the electrode.

Lithiophilic coating is an effective strategy to enhance the wettability between liquefied lithium and carbon matrix, because these coating layers can react with molten lithium to reduce the surface energy of carbon matrix or form alloys with lithium. Silicon can coat on the carbon-based materials via chemical vapor deposition to form the Li_x_Si alloys (Liang et al., 2016; Hapuarachchi et al., 2018). Thus, the molten metallic lithium can easily and quickly flow into the scaffolds with silicon coating. Some metal oxides, such as ZnO (Zhao et al., 2019; Yue et al., 2020), CuO (Wu et al., 2018; Zhang C. et al., 2018; Huang K. et al., 2019), Co_3_O_4_ (Li S. Y. et al., 2019; Pan et al., 2019), can undergo redox reactions with molten lithium. Based on this fact, the scaffold coated with metal oxide can obtain enhanced lithiophilicity. Yue et al. coated Cu_x_O (CFeltCu) on a porous carbon felt (CFelt) and prepared a stable CFeltCu-Li composite anode via thermal infusion method (Yue et al., 2019a). During the thermal infusion process, the Cu nanoparticles derived from copper oxide reduced by molten lithium are evenly dispersed on the surface of the CFelt. To some extent, these Cu nanoparticles with high conductivity can not only regulate the Li stripping/plating behavior, but also reduce the local current density of anode. Therefore, the CFeltCu-Li composite anode presented outstanding cycle stability (over 1,000 h) and low overpotential (25 mV) without dendrite growth in symmetric cells.

**Figure 2:**
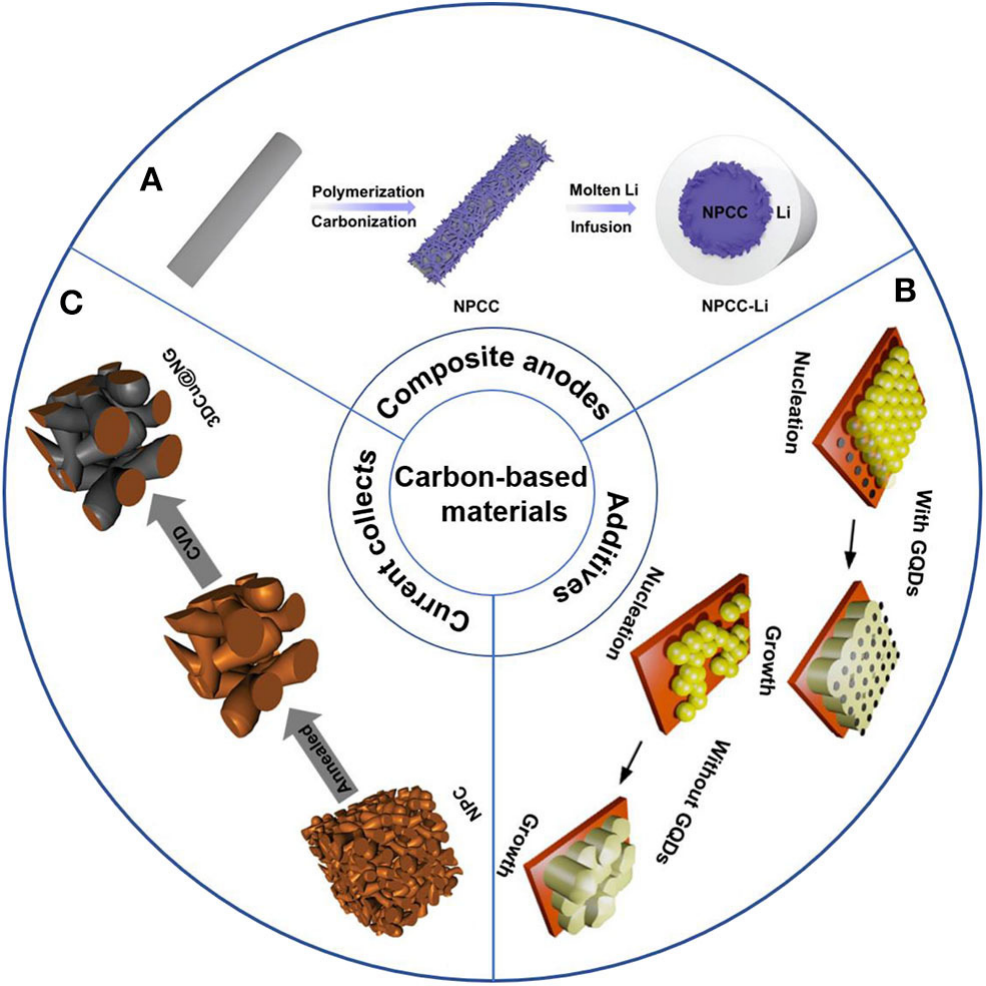
Schematic diagram of the different strategies of carbon-based materials in addressing the challenges of lithium metal anodes: **(A)** Scheme illustration of the synthesis process of NPCC-Li. Li C. et al. (2019) with permission from WILEY-VCH. **(B)** Illustration of the GQDs regulated deposition processes Reprinted with permission from Hu Z. et al. (2020) with permission from Elsevier. **(C)** Schematic illustrations of the fabrication of 3D Cu@N-doped graphene. Reprinted with permission from Zhang Z. et al. (2018a) with permission from WILEY-VCH.

Although the metal oxide coating can significantly improve the lithium affinity of carbon-based materials, the specific capacity and rate performance of lithium anodes have deteriorated to some extent. Carbon matrix with heteroatom co-doping can effectively overcome the above mentioned obstacle. A stable lithium composite anode that is composed of N and P co-doped carbon cloth and lithium metal, was presented by Li and coworkers (Figure 2A) (Li K. et al., 2019). N and P can provide enhanced surface lithiopholicity for carbon-based material, facilitating molten lithium diffusion and uniform coating. This composite anode delivered stable voltage hysteresis over 600 h at a current density of 3 mA cm^−2^.

All the aforesaid approaches require complex preparation processes. In order to simplify the synthesis process, Go et al. made carbon cloth more affinity between lithium and carbon by a facile heat treatment in air (Go et al., 2019). Numerous nanocrevasses can be introduced in carbon cloth during the heat treatment, allowing the successful infusion of molten lithium. As a result, the as-prepared composite anode with lower local current density presents long-term cycling and dendrite-free morphology.

### Carbon-Based Materials as Additive

Liquid organic electrolytes play a vital role in LIBs, due to their good wettability with electrode and ionic conductivity. However, the side reaction between lithium metal and electrolyte has negative affect on the electrochemical performance of lithium metal anode. The liquid electrolyte is composed of solvent, lithium salt and additives, which determine the uniformity and stability of SEI film. Therefore, modifying the additives can improve the performance of the electrolyte, then changing the deposition morphology of lithium (Tao et al., 2017; Wang et al., 2020).

In general, soluble Li-containing compounds and organic compounds, such as LiNO_3_ (Yan et al., 2018), LiF (Wang et al., 2019) and fluoroethylene carbonate (FEC) (Zhang R. et al., 2017), are commonly employed as additives. These additives are generally used as sacrifices to react with lithium metal in advance, forming a SEI film with controllable composition and good stability. On the contrary, when the carbon-based material is used as additive, it serves as the initial nucleation site for Li deposition instead of reacting with Li. Nanodiamond with a size of ~ 5 nm and high crystallinity is an early member of the carbon-based material family (Cheng et al., 2017). The nanodiamond particles treated by octadecylamine (ODA) can be well-dispersed in the ester-based electrolyte. In Li||Cu half-cell batteries, due to the nanodiamonds inherit large surface area and strong binding energy with Li, the Li^+^ can adsorb on the surface as the initial heterogeneous seeds instead of growing on the copper current collector. Moreover, nanodiamond-guided Li deposits are small enough to form a uniform distribution of deposition. When charging, the co-deposits of nanodiamond and Li can be stripped into the electrolyte to maintain a stable content of nanodiamond in the electrolyte, improving the cycling stability of the Li anode. Whereas, the nanodiamonds tend to aggregate and form clusters easily, which is negatively affect the long-term cycle.

With the development of technology, more and more new materials are recognized and applied in various fields. Graphene quantum dots (GQDs) with tiny size can be well-dispersed in the electrolyte without further modification (Deng et al., 2016; Park et al., 2016; Tam et al., 2019). Hu et al. directly added GQDs to the electrolyte, which is composed of Li bis (trifluoromethane)-sulfonimide (LiTFSI, 1.0 M), 2.0 wt% LiNO_3_, 1,3-dioxolane (DOL) and 1,2-dimethoxyethane (DME; 1:1 by volume) (Hu Y. et al., 2020). Due to the quantum confinement effect (Zhang W. et al., 2018), GQDs act as the heterogeneous seeds enable continuously adjust ion dispersion and avoid high local electric field in the subsequent plating process, which is conducive to the uniform deposition and inhibit the dendrite growth (Figure 2B). However, the complex synthesis procedures and extreme conditions of GQDs leads to high costs, which limits its large-scale application.

### Carbon-Based Materials as Current Collect

The current collector is one of the important components of LIBs. It is commonly used as a substrate to support active materials. During battery operation, the current collector not only transfer electrons between the active material and the external circuit, but also diffuse the heat generated inside the battery (Jin et al., 2018; Zhang Z. et al., 2018b). Generally, planar copper foil is used as anode current collector, while it is easy to cause severe dendrite formation. Due to the porosity, low cost, good electronic conductivity and confinement of 3D current collectors, they have attracted great attention from researchers. 3D metallic materials, such as copper foam, nickel foam and aluminum foam, with good electronic conductivity and high specific surface area have been regarded as the most competitive candidates. However, the nucleation overpotential of lithium deposited on the 3D metal structure is relatively higher than other current collectors, causing uneven nucleation and inhomogeneous lithium deposition. Carbon-based materials have good lithium affinity, and composite with metal materials can effectively improve battery performance. When N-doped graphene combined with 3D metal materials, an improving LMBs performance can be achieved. Zhang et al. prepared a 3D porous copper coated with N-doped graphene via a CVD process (Figure 2C) (Zhang R. et al., 2018b). Due to the presence of pyridinic and pyrrolic nitrogen, there is a strong interaction between N-doped graphene and Li^+^, leading to homogeneous Li^+^ flux and a uniform Li deposition.

The morphology of carbon-based material is critical to control the lithium loading and deposition location. The 3D carbon-based materials with random sponge-like structure lead to Li^+^ preferentially deposit on the outer surface of the 3D framework. To solve this problem, a sequence of materials with regular structures have been investigated. A 3D construction is fabricated by vertically arranged nanofibers (VACNFs) directly grown on a planar copper foil. When it acts as a host for the lithium metal anode (Chen Y. et al., 2019), the special structure of VACNFs not only provides well-aligned brush-like space for Li^+^ deposition, but also enhances the surface electrochemical activity because of the active graphitic edge sites. Therefore, this composite material can effectively decrease the local current density, suppress the lithium dendrite growth and result in dendrite-free Li deposits.

Although the 3D metal structure exhibits excellent performance as a current collector, the defects, such as high density and easy erosion, cannot meet the requirements of high-performance storage systems. Compared with active materials, the density of metal current collectors is generally higher, resulting in a low mass proportion of active materials in the entire electrode, which inhibits the improvement of the energy density of the entire battery (Zhou et al., 2018). During the repeated charge/discharge process, the metal current collector electrochemically eroded, resulting in a short cycle life. Carbon-based materials with light weight and good chemical stability can avoid the aforementioned problems encountered by metal current collectors. Among carbon-based materials, carbon nanotubes (CNTs) are highly conductive and commercially available, and as an electrode behaving good lithium storage capacity (Che et al., 1998). Yang et al. used the commercial CNTs sponge as a current collector, which has high specific surface area and graphitic-amorphous carbon composite feature (Yang G. et al., 2019). In the initial stage of electroplating, the lithium-storage of the CNTs (above 0 V) makes it a “pre-lithiated” host, enhancing its wettability with subsequent lithium deposits (below 0 V) and lowering the lithium nucleation overpotential. More importantly, the high surface area of the porous CNTs sponge enable the increased density of lithium nucleation sites and reduced local current density on the carbon nanotubes as well as uniform lithium deposition.

## Conclusions

In recent decades, researchers have been committed to developing more strategies to meet the safety and high energy storage requirements of lithium metal anodes. Carbon-based materials with various structures and unique chemical properties play a significant role in minimizing the shortcomings of lithium metal anodes. This review outlines the challenges of lithium metal anodes and the diverse strategies of carbon-based materials in advanced LMBs.

The diversity of carbon-based materials makes it play a specific role in different strategies to solve the safety issues of lithium metal anodes. Nanodiamonds and GQDs with a size of several nanometers can be used as electrolyte additives to form initial nucleation sites, guiding the uniform deposition of Li^+^ on the electrode surface. As the size of the carbon-based material increases, it has greater flexibility, higher porosity and a larger surface area, which helps it be modified or composited with other materials. The abundant functional groups on the surface of the carbon-based material enable it combine with other metal oxides or as a coating material for other 3D porous frameworks. The 3D porous scaffolds composite constructed with lithiophilic materials can be used as a current collector for Li^+^ deposition and be assembled with lithium metal to form a composite electrode. Therefore, the 3D porous scaffolds can effectively overcome the “hostless” problem of lithium metal, accommodate huge electrode volume changes during electrochemical process, as well as contribute to a stable cycle life.

On account of the special structural and morphological features, carbon-based materials are also widely used in other research fields. Carbon-based materials can not only be employed as the electrode materials for LIBs and supercapacitors, but also act as the metal-free electrocatalysts for oxygen reduction reaction, oxygen evolution reaction, and hydrogen evolution reaction, because of its large specific surface area, defective sites, as well as tunable electronic structure.

Carbon-based materials with low price, abundant nature reserves, versatile structure easy fabrication, have a significant impact in the field of electrocatalysis and energy storage, especially for large-scale high-energy-density batteries. It is believed that the development of carbon-based materials plays a significant role in the commercial application of LMBs.

## Author Contributions

All authors listed have made a substantial, direct and intellectual contribution to the work, and approved it for publication.

## Conflict of Interest

The authors declare that the research was conducted in the absence of any commercial or financial relationships that could be construed as a potential conflict of interest.
